# Assessing visuospatial perception in clinical and healthy populations: Test–retest reliability and smallest real difference of hill steepness estimation and the distance-on-hill task in virtual reality

**DOI:** 10.1007/s00426-025-02125-0

**Published:** 2025-05-20

**Authors:** Erin MacIntyre, Mirinda M. Whitaker, Felicity A. Braithwaite, Jeanine K. Stefanucci, Tasha R. Stanton

**Affiliations:** 1 IIMPACT in Health, Allied Health and Human Performance, University of South Australia, GPO Box 2471, Adelaide, South Australia 5001 Australia; 2 Persistent Pain Research Group, Hopwood Centre for Neurobiology, Lifelong Health Theme, South Australia Health and Medical Research Institute (SAHMRI), Adelaide, Australia; 3 Department of Psychology, University of Utah, Salt Lake City, Utah USA

## Abstract

Theories of embodied perception posit that the visuospatial perception of one’s environment is not only bound by features of the environmental itself, but also by the body capacity and affective state of the individual. Hill steepness and distance estimation tasks are widely used to assess the influence of physiological and psychological factors on visuospatial perception, but their test–retest reliability and measurement error are unknown. Such information is important to contextualise repeated measures study designs and understanding individual level differences. We aimed to evaluate the test–retest reliability and establish the smallest real difference (SRD) of three commonly used visuospatial perception tasks (ascending and descending hill steepness estimation, the distance-on-hill task) in healthy controls (*n* = 33) and people with painful knee osteoarthritis (*n* = 33). All participants completed the virtual reality visuospatial perception tasks two times, one week apart. Intraclass correlation coefficients (ICC), Bland–Altman plots, and SRD were used to evaluate the tasks. Our results revealed that both hill steepness estimation tasks are suitable for repeat administration in both populations given excellent reliability (uphill ICC = 0.80 to 0.85; downhill ICC = 0.89 to 0.90) and high sensitivity to change (uphill SRD = 17.7 to 18.9 degrees; downhill SRD = 12.1 to 14.7 degrees). The distance-on-hill task may have limited utility due to its poor reliability (ICC = 0.29 to 0.38) and low sensitivity to change (SRD = 6.20 to 8.5 m). Our findings provide methodological support for the use of hill steepness tasks as a measure of visuospatial perception in embodied perception research.

**Supplementary Information:**

The online version contains supplementary material available at 10.1007/s00426-025-02125-0.

## Introduction

Contemporary perceptual theory posits that perception is embodied and enactive. That is, perception is bound by one’s current bodily state (Proffitt, 2006) and can be action-specific, or influenced by one’s capacity to act within an environment (Witt et al., 2004). Modifiable by physiological, social, and affective factors (Proffitt et al., 2003), visuospatial perception is thought to be adaptive, acting as a “biological ruler” which scales the world based upon one's current capacity (Proffitt et al., 2022). When capacity is low, such as when an individual is fatigued, the world is viewed as harsher – distances are perceived as farther and hills as steeper – making us more likely to avoid acting within these environments (Proffitt, 2006). When capacity is high, the reverse occurs (Schnall et al., 2010), potentially making us more likely to engage with an environment. Such perceptual scaling may hold relevance to populations in whom activity avoidance is high, such as in people with persistent pain (Volders et al., 2015). People with persistent pain overestimate distance relative to pain-free controls (Witt et al., 2009), with pain-induced visuospatial perception scaling showing specificity to action – i.e., occurring only when participants are allowed to move (Tabor et al., 2013). Similarly, those with phobias may visually exaggerate a threat, whereby feared objects are perceived as larger and/or closer, which can lead to an increase in avoidance or safety behaviours (Givon‐Benjio et al., 2020; Vasey et al., 2012). Accordingly, there is growing interest in using visuospatial perception tasks within clinical populations as a treatment target or a moderator of clinical effect, although, to date, results have been mixed (Dreyer-Oren et al., 2019; Li & Graham, 2021; Malighetti et al., 2020). Interpretation of null results, in particular, is difficult given that the measurement properties of visuospatial perception measures are relatively unknown.

Visuospatial scaling has been assessed in numerous and varying ways. Common tasks include assessment of perceived hill steepness of ascending and descending slopes or distance estimation of flat surfaces, gap widths, and heights. For hill steepness and distance estimation tasks, the error between the estimated and actual stimuli is calculated, with the magnitude and direction of error thought to reflect alterations in visuospatial perception (e.g., a positive error indicates overestimation of steepness/distance). Additionally, more nuanced measures of perceptual bias are common, such as the distance-on-hill task. In this task, participants estimate the distance to a target that is presented on a flat surface and on a hill, where the *actual* distance to the target is the same in both instances (Laitin et al., 2019a, b; Stefanucci et al., 2005). Embodied perception posits that the effort taken to traverse a distance on a hill is greater than the same distance on flat; thus, people will display a perceptual bias whereby they perceive the same distance as farther on a hill than on flat ground.

These perceptual tasks have been used widely to evaluate the effect of differing bodily states, including fatigue, age, weighted backpacks, body mass index, low blood glucose (Bhalla & Proffitt, 1999; Cole & Balcetis, 2013; Sugovic et al., 2016; Taylor-Covill & Eves, 2013) as well as the effect of threat on visuospatial perception, in both clinical (Clerkin et al., 2008; Givon‐Benjio et al., 2020) and non-clinical populations (Givon-Benjio & Okon-Singer, 2020; Stefanucci et al., 2008). Most studies find that bodily state and threat bias visuospatial perception in a manner consistent with theories of embodied perception (i.e., visuospatial overestimation is related to decreased capacity or increased threat), although results sometimes conflict depending on the task and environment (Dean et al., 2016; Durgin et al., 2012; Keric & Sebanz, 2021). Several factors have been identified in the literature to explain these conflicting results, including controversy surrounding what these tests capture (e.g., task-related demands versus true changes in visuospatial perception) (Durgin et al., 2009, 2012), and the unknown psychometric properties of visuospatial perception tasks themselves (Firestone, 2013; MacIntyre et al., 2022; Philbeck & Witt, 2015).

Despite this controversy and repeated calls to assess the psychometric properties of visuospatial perception tasks, few studies have evaluated task reliability. Such knowledge is critical given that studies evaluating perceptual shifts in experimental or clinical trials often require repeated-measures designs. Our recent systematic review investigating the influence of bodily state on visuospatial perception of the environment (MacIntyre et al., 2022) found that nearly one third (19/68) of studies assessed visuospatial perception across multiple timepoints. Thus, at present, differences in visuospatial perception between timepoints assumed to reflect experimental manipulation cannot be delineated from unreliable test performance, learning or adjustment to the task itself, or measurement error of the test. To the authors’ knowledge, there have been no published studies that evaluate the reliability of hill steepness estimation tasks, and only one study has evaluated reliability of the distance-on-hill task. Specifically, Laitin et al., (2019a) found excellent intra-subject reliability for a virtual distance-on-hill task between the start and end of a single testing session (Spearman-Brown coefficient = 0.93). However, the stability of responses over multiple sessions separated in time remains unknown. The current study fills this gap by evaluating test–retest reliability of both hill steepness estimation and the distance-on-hill task for the first time.

To make our reliability findings relevant to clinical audiences, we also calculated the smallest real difference (SRD) of the perceptual tasks. The SRD is a measure of precision of a test, allowing determination of whether an individual’s change in test performance over time is greater than an index of measurement error of the test itself, within a 95% confidence interval (Wier et al., 2006). The SRD helps inform clinical significance, or to what degree a change in a measure over time is *meaningful,* beyond expected measurement error of a given assessment. Given preliminary evidence that interventions may reduce the degree of perceptual overestimation observed in clinical conditions (Dreyer-Oren et al., 2019; Shiban et al., 2016), understanding the SRD will aid in interpretation of results. For example, biased visuospatial perception has been observed in those with social anxiety disorder (Givon‐Benjio et al., 2020), and arachnophobia (Li & Graham, 2021). If the change in visuospatial perception over time exceeds the SRD of the perceptual task, it would likely reflect a true change in perceptual task performance (and one with possible clinical relevance), rather than reflecting noise inherent in repeated measurements or just more experience with the task. Such scores provide critical information for the clinical interpretation of results and the understanding of individual differences in visuospatial perception over time.

Here, we aimed to evaluate the test–retest reliability and SRD of three common visuospatial perception measures (uphill steepness estimation, downhill steepness estimation, and distance-on-hill) in two populations – a healthy control group and people with painful knee osteoarthritis (OA). It is well established that psychometric evaluation requires population specificity. Thus, we aimed to evaluate visuospatial perception in both a healthy control group as well as a population for which altered perceptions of the surrounding environmental harshness may hold high clinical relevance. Our past work has shown that people with painful knee OA perceive hills as steeper than age- and gender-matched healthy controls (MacIntyre et al., 2024). Without understanding the reliability of such tasks in both painful and non-painful populations, it is uncertain whether this finding represents a true difference between groups or whether it is likely measurement error. Given that nine in ten people with painful knee OA are physically inactive (Wallis et al., 2013), despite recommendations otherwise (Bannuru et al., 2019), understanding if these altered perceptions of the environment result in activity avoidance is key and would uncover a novel clinical target to facilitate more physical activity. Evaluating causal links between altered environmental perception and physical activity requires longitudinal studies; thus, an essential first step is to understand the test–retest reliability of visuospatial perception measures.

## Methods

This study received ethical approval from the University of South Australia Human Research Ethics Committee (No. 203778 and No. 204598 for pain-free controls and knee OA groups respectively) prior to data collection. The study and analysis plan were pre-registered via a time-locked protocol on Open Science Framework (https://osf.io/btfsh/), however deviations from this plan occurred and are outlined below. This manuscript is reported in accordance to the Guideline for Reporting Reliability and Agreement Studies (GRRAS) (Kottner et al., 2011). See Supplementary File 1 for the completed reporting checklist.

### Participants

Two groups were recruited: a healthy control group and a persistent pain group of participants with knee OA. Based on an a priori sample size calculation (ICC = 0.4 allowing detection of poor task reliability, power = 80%, alpha = 0.05, at two timepoints), 36 participants were required in each group (Bujang & Baharum, 2017). Given potential clinical relevance of these visuospatial perception tasks, our sample size calculation prioritised avoidance of Type I error; i.e., incorrectly classifying our tasks as being reliable when they are not. Convenience sampling via electronic advertisements, word of mouth, existing participant databases, and posters at local community centres was used to recruit participants. All participants provided written informed consent prior to the start of the experiment and received $40 AUD after completing both testing sessions.

The healthy control group participants were recruited as part of a larger experimental study (MacIntyre et al., 2024). This larger study evaluated between-group differences in visuospatial perception using the same tasks described here. The first 35 consecutive healthy control group participants in the previous study were invited to participate in the current study, which involved attending a second identical session to evaluate task test–retest reliability (further details below). Participants were > 50 years old (to match the knee OA group), had no current pain (anywhere in the body), no history of a significant pain disorder (via self-report of a pain condition that lasted > 3 months), no neurological disorders, and had normal or corrected-to-normal vision.

The knee OA group participants were recruited as part of two experimental studies (each with two in-person testing sessions) evaluating the feasibility/acceptability of novel technologies to promote exercise engagement. In both experimental studies, knee OA participants experienced the novel technology and completed a single, brief (≤ 30 min) bout of self-paced exercise (cycling or treadmill walking). Participants were able to stop exercising at any point in time, and their knee pain levels were monitored closely (i.e., exercise was ceased if pain intensity increased by > 20pts on a 0–100 NRS during at any point during the exercise session). A subgroup of these participants was invited to participate in the current experiment until the sample size was met. In all cases, knee OA participants completed the current visuospatial perception tasks at the beginning of each session, prior to experiencing the technology/completing exercise tasks. Knee OA participants who had stable pain across both sessions (≤ 2pt difference in reported average pain intensity over the previous week on a 11pt NRS) were included in the main analysis. Those with unstable pain still completed the tasks during the second session and were included in a secondary analysis. All participants in the knee OA group met the National Institute for Health Care and Excellence (NICE) clinical criteria for knee OA (National Clinical Guideline, 2014). Additionally, knee OA participants were required to experience moderate levels of pain (average knee pain over the previous week ≥ 4 on an 11pt NRS) and did not have comorbidities that prevented safe participation in exercise. We did not pre-register reliability evaluation in people with knee OA, but the pre-registered methods and analysis for the healthy control group were replicated in the knee OA group.

### Study procedures

Participants in both groups attended two in-person data collection sessions, approximately one week apart. In both sessions, immediately prior to the visuospatial perception tasks participants completed paper or electronic surveys (REDCap), which collected process variables that have been found to influence visuospatial perception (detailed below in outcome section). Next, participants underwent the three visuospatial perception tasks in a sequential order (uphill steepness, downhill steepness, and distance-on-hill estimation) in virtual reality (VR) using a head-mounted display (healthy control group used an HTC Vive; New Taipei City, Taiwan and knee OA group used Oculus Quest; Melno Park, CA). Custom VR software was developed using Unity Software (San Francisco, CA) and Steam VR (Valve Corporation, Bellevue, WA). All participants completed the visuospatial perception tasks under the guidance of a single researcher (EM).

**Fig. 1:**
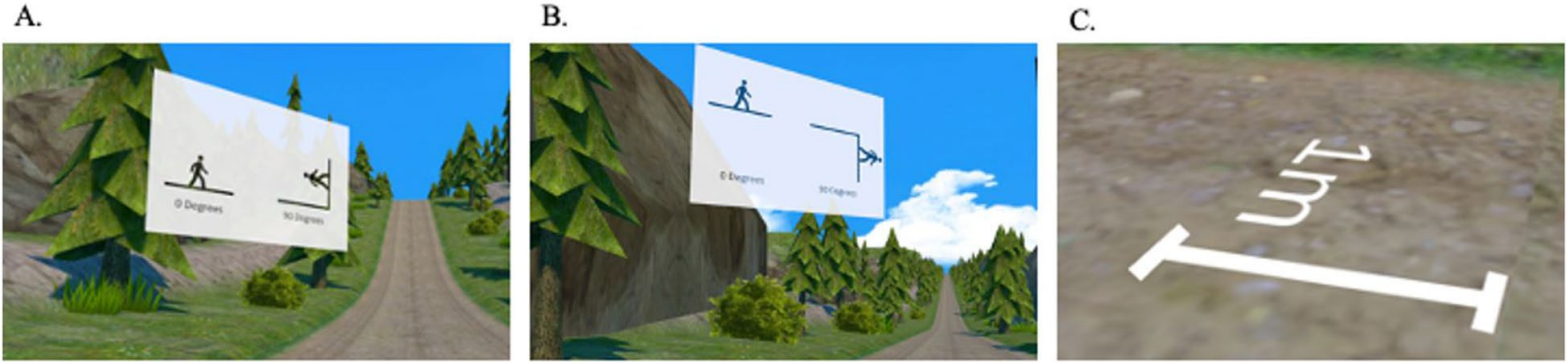
Depiction of visual anchors for visuospatial perception tasks. **A** Uphill steepness (0 degrees and 90 degrees). **B** Downhill steepness (0 degrees and 90 degrees). **C** Distance estimation (1 meter)

During the VR tasks, participants were in a standing position and placed their hands on top of a table while using the VR to reduce their risk of falling. First, participants underwent familiarisation with the virtual world, where they were instructed to visually explore the environment by turning their head and trunk, while remaining in the same spot with their hands on the tabletop. Prior to the hill steepness estimation (uphill and downhill), participants were given a verbal explanation of angles (e.g., 0 degrees is flat and 90 degrees is vertical), and were provided with visual anchors (i.e., what 0 degrees and 90 degrees looks like). These anchors were visually displayed for 3 practice hills (Fig. 1A and B). Following familiarisation with hill steepness estimation judgements, participants began formal testing of the perceptual tasks. For both hill steepness tasks, participants were presented 20 hills in a randomised order. The hills were one of five differing slopes (5 degrees, 10 degrees, 15 degrees, 20 degrees, or 25 degrees) and two lengths (54 m or 154 m). Participants verbally estimated perceived hill steepness. During the downhill steepness task, participants first estimated hill steepness followed by verbally rating their state fear of walking down the hill using the Subjective Units of Distress Scale (0–100, where 0 = no fear, and 100 = worst fear imaginable) (Cohen et al., 1983). Otherwise, the procedure for the ascending and descending hill steepness tasks was identical, the only difference being the participants’ location in the virtual world (i.e., standing at the bottom or the top of the virtual hill). Next, the participants completed the distance-on-hill task. In this task, participants verbally estimated the distance to five targets (11 meters, 14 meters, 18 meters, 21 meters, 26 meters), on both a flat surface and on a 20 degree hill, using a virtual cone placed at each of the target distances. Prior to formal distance estimations, there was a single practice round, during which a virtual ruler (1 meter or 3 feet 3 inches, Fig. 1C) was displayed to give a visual representation of the units of distance to be used to verbally report distances in the virtual environment. Following this, participants estimated distances. The task order (flat vs. hill) and the order of target distances within each task were both randomised. Participants could estimate distance in either feet or metres, whichever units they were most comfortable with. All participants’ verbal hill steepness and distance estimations were manually entered into the VR program by the researcher.

### Primary outcomes

The primary outcomes were mean ascending hill steepness error, mean descending hill steepness error, and mean distance-on-hill error.

For hill steepness measures, error was calculated as the difference between the actual and reported hill steepness, where a positive value indicates an *overestimation* of hill steepness, and a negative value indicates *underestimation*. The mean of all ascending hill steepness errors was calculated as was the mean of all descending hill steepness errors. Consistent with our protocol, we also calculated mean hill steepness error for shallow (mean of 5 degree and 10 degree hill error) and steep (mean of 20 degree and 25 degree hill error) hills, given that past research has indicated an effect of hill steepness on estimations (i.e., that overestimation increases as actual hill steepness increases) (Bhalla & Proffitt, 1999; Durgin & Li, 2017).

For the distance-on-hill measure, the difference between the distance estimation for each target distance on the hill relative to the flat was calculated, where a positive number indicates that the target distance on the hill was reported to be *farther* than the same target distance on the flat. The mean distance estimate error (difference between hill vs flat) was calculated across all target distances.

### Process outcomes

Several physiological and psychological states have been found to influence performance of visuospatial perception tasks (Molto et al., 2020; Schnall, 2017). As such, we evaluated the following known influential variables at both sessions: current fatigue (7 point Numerical Rating Scale, where 0 = no fatigue, and 6 = extremely fatigued); depression (4-item PROMIS depression subscale) (Cella et al., 2019); state anxiety (4-item PROMIS anxiety subscale); stress (4-item Perceived Stress Scale; healthy group only) (Cohen et al., 1983); general mood (11pt NRS, where 0 = very bad, as depressed as I could be and 10 = excellent; knee OA group only) (Mouatt et al., 2023) and the time of day of testing. For each participant, we aimed to schedule their two sessions at the same time of day and asked them to maintain their normal routine (e.g., waking at similar time on both days, eating at a similar time). Participants in the healthy control group also completed the iGroup Presence Questionnaire, which evaluates the experience of ‘being there’, on three domains: presence (being physically present in the virtual space); involvement (awareness of only the virtual world and isolation from the ‘real’ world); and realism (how realistic the virtual world is).

### Statistical analysis

First, process variables were assessed to determine clinical stability across sessions. Normality of process variables were first assessed via Shapiro–Wilk test and by visually inspecting the residuals via QQ-plots. In cases where residuals were normally distributed, paired t-tests were conducted to compare session 1 and 2 scores, otherwise non-parametric Mann–Whitney U tests were used. The groups were considered clinically stable if there was no statistically significant difference detected in process variables between sessions (given alpha = 0.05). To evaluate the potential presence of learning effects, we performed and off-protocol analysis to compare the hill steepness estimation errors in each group were compared between sessions using a 2 (group) × 2 (session) × 5 (hill steepness magnitude) RM ANOVA.

To evaluate test re-test reliability, intraclass correlation coefficients (ICC’s, two-way mixed effects model, average measures) were calculated in SPSS Statistics v28.1 (IMB, Chicago, Illinois) to assess the absolute agreement between the two sessions for the three visuospatial perception measures (Koo & Li, 2016; McGraw & Wong, 1996). This ICC formula was chosen according to study aims and methods (i.e., accounting for multiple measurements of a visuospatial perception task using a single rater) and is consistent with McGraw and Wong’s (1996) classification of ICC models. The formula for this ICC model is: $ICC=\frac{{MS}_{R} -{MS}_{E}}{{MS}_{R} +\frac{{MS}_{R}- {MS}_{E}}{n}}$ (Koo & Li, 2016), where MS_R_ = mean square for rows, MS_E_ = mean square for error, and n = number of subjects. According to our protocol, the ICC was to be performed in jamovi (Jamovi Software, 2022) using the seolmatrix (Seol, 2021) module; however, this software did not provide sufficient information regarding the model (e.g., two-way vs. one-way effects), therefore the analysis was run in SPSS. Based on recommendations from Koo and Li (2016), ICC values below 0.5 indicate poor reliability, values from 0.5 to 0.75 indicate moderate reliability, values from 0.76 to 0.90 indicate good reliability, and values above 0.90 indicate excellent reliability. Bland–Altman plots were created to visually inspect the between session ratings for all visuospatial perception measures.

As a preliminary indication of clinical significance, the Smallest Real Difference (SRD) was calculated for each visuospatial perception measure. The SRD is an individual measure of the sensitivity of change of a test and provides information about the threshold at which an individual’s score exceeds the assumed error at the 95% confidence level (Beckerman et al., 2001). The SRD is an important metric for single-subject applications (e.g., prescribing an intervention based on visuospatial perception task performance), and individual difference research. Given the potential clinical implications of this research, we felt it was worthwhile to have a metric of clinical significance and chose the SRD as it is commonly used metric in clinical practice (Weir, 2005). The SRD was calculated using the formula: $SRD=1.96 \times SEM \times \surd 2$, where the SEM is the square root of the error term from the ICC ANOVA. This method has two advantages: 1) it does not vary depending on the ICC model used; 2) it is not sensitive to between-subjects variability, and therefore largely independent from the population it was derived from (Wier et al., 2006). The SRD analyses were not pre-registered but were included to provide enhanced context and clinical utility to the results.

To confirm that our results were not impacted by our statistical exclusion criteria (i.e., requiring stable clinical presentation), we also conducted a secondary analysis, which included all participants with knee OA, including those whose knee pain was not stable between sessions (i.e., > 2pt between-session difference in pain intensity on 11pt NRS).

To supplement the frequentist results, Bayesian models were run in cases of null results. Bayesian models were run in R version 4.3.3 (R Core Team, 2024); using the rstan (Stan Development Team, 2024) and brms (Bürkner, 2018) packages. Betas, credible intervals, estimated errors (EE), and Bayes factors (BF)^1^ are reported for all Bayesian analyses. Bayesian credible intervals are analogs to frequentist confidence intervals that summarize the posterior distribution and provide a probability statement that an unobserved true parameter would lie within an interval a certain percent (e.g., 90, 95, 99) of the time, given the observed data. For example, a 95% credible interval provides a probability statement that given the observed data, the unobserved true parameter would fall within the given interval 95% of the time. Bayes factors are used to compare to hypotheses/models and are mathematically defined by dividing the likelihood of data under one hypothesis/model by the likelihood of the data under another hypothesis/model, with a Bayes factor of 1 suggesting that the data are equally likely under either hypothesis/model. All Bayesian analyses were run with the default priors in brms, which are flat/uninformative for all parameters of interest. It is worth noting that Bayes factors are sensitive to prior specification (Aitkin, 1991; Gelman & Shalizi, 2013; Liu & Aitkin, 2008), so the Bayes factors presented should be interpreted with this in mind. Given this, our interpretation of the Bayesian analyses focuses more on the information provided by the credible interval, as the posterior distribution summarized by the credible interval is more stable and less sensitive to the prior than Bayes factors especially as sample size increases.

## Results

We recruited 35 participants for the healthy control group, and 36 participants for the knee OA group. Both groups were older (healthy control group = 66.8 years [SD = 6.59], knee OA group = 66.0 years [SD = 8.58]), and predominantly female (healthy control group *n* = 23 female, knee OA group *n* = 19 female). Retention was high; across both groups all but one participant completed both sessions. Two participants were excluded from the healthy control group, one due to not attending the second session, and the other due to diagnosed knee OA (no current pain). Three participants in the knee OA group were excluded from the primary analysis due to unstable knee pain. The results for our secondary analysis of the full knee OA sample, including those with unstable pain, are provided in Supplementary File 3. The mean time between sessions was 10.3 days (SD = 6.77 days) for the healthy control group, and 9.58 days (SD = 5.77) for the knee OA group. Participant demographics and process outcomes for both sessions are presented in Table 1. There were no significant differences (*p* < 0.05) in process outcomes between Session 1 and 2, except in the knee OA group, where there was a small decrease in pain scores between sessions (mean difference = −0.45, 95% CI −0.86 to −0.06), and the time of the session in the knee OA group, which was 31 min earlier in session 1. Bayesian results for process variables were largely consistent with frequentist results (see Table 1). In some cases, the BF suggested that the model with session included (e.g., state fear during downhill steepness estimation), was 10–20 times more probable than the model without session included. However, the credible intervals were wide, and in all but one case (VR realism) included 0. Given the sensitivity of BF to prior specification, and the lack of evidence for an effect from the credible intervals, we did not include any process variables as covariates in subsequent analyses.

**Table 1:** Participant characteristics and process variables for both sessions

			Between session differences Frequentist				Between session differences Bayesian		
	Session 1 Mean (SD)	Session 2 Mean (SD)	*p*-value	Cohen’s *d*	Mean difference, 95% CI		Beta	Bayes Factor	Estimated Error, 95% CrI
Healthy Control Group									
Age	66.8 (6.59)								
Gender	23 female, 10 male								
Time of session	11:10 AM (1.63 h)	11:09 AM (1.64 h)	0.96	−0.05		−0:01, −0.43 to 0.41			
Fatigue	1.18 (1.13)	1.55 (1.18)	0.27	0.32		0.28 −0.23 to 0.78	0.37	1.63	0.30 −0.23 to 0.95
Anxiety	6.25 (2.69)	6.47 (3.02)	0.75	−0.06		−0.22 −0.40 to 0.29	0.21	1.88	0.71 −1.14 to 1.61
Depression ^a^	5.91 (2.97)	5.91 (3.26)	0.86	-		0.00	0.00	1.95	0.80 −1.52 to 1.57
Perceived Stress ^a^	3.59 (2.55)	3.63 (2.54)	0.86	-		0.04	0.03	1.60	0.64 −1.21 to 1.30
Average state fear ^a^	6.41 (14.2)	6.85 (15.8)	0.57	-		0.44	0.44	5.46	2.17 −3.82 to 4.72
25 deg hills state fear^a^	17.6 (21.1)	19.5 (24.4)	0.53	-		1.90	1.88	14.2	5.50 −9.03 to 12.77
VR presence^a^	3.26 (1.02)	3.21 (1.04)	0.98	-		−0.05	−0.05	0.67	0.26 −0.56 to 0.46
VR realism	2.46 (0.70)	2.28 (0.75)	0.22	0.25		0.18 −0.11 to 0.48	−0.18	0.76	0.07 0.62 to 0.88
VR involvement	3.23 (1.40)	2.93 (0.97)	0.25	0.25		0.30 −0.22 to 0.82	−0.30	1.24	0.31 −0.91 to 0.29
Knee OA group									
Age	66.0 (8.58)								
Gender (count)	19 female, 14 male								
Bilateral knee pain (count)	24								
Time of session	10:46 AM (1.58 h)	10:14 AM (1.69 h)	0.02*	0.44		−0.53 −0.95 to −0.10			
Fatigue	1.91 (1.16)	1.55 (1.28)	0.06	0.30		−0.37 −0.72 to 0.01	−0.37	1.58	−0.31 −0.98 to 0.24
Anxiety ^a^	5.91 (2.47)	5.70 (2.36)	0.41	-		−0.21	−0.21	1.60	−0.60 −1.40 to 0.96
Depression ^a^	6.03 (3.26)	5.82 (2.88)	0.61	-		−0.21	−0.22	1.98	−0.77 −1.71 to 1.30
Mood	8.00 (1.73)	7.79 (1.82)	0.30	0.12		−0.21 −0.63 to 0.20	−0.21	1.24	−0.44 −1.07 to 0.65
Average state fear	25.8 (19.9)	23.9 (15.5)	0.36	0.10		−1.83 −5.88 to 2.22	−0.68	11.4	−4.57 −9.55 to 8.27
25 deg hills state fear	52.5 (34.0)	53.9 (30.2)	0.69	0.04		1.34 −5.44 to 8.11	1.50	20.23	8.16 −14.3 to 17.9
Pain intensity	5.21 (1.83)	4.76 (1.80)	0.03*	0.25		−0.45 −0.85 to −0.06	−0.46	1.89	−0.45 −1.34 to 0.42

Results are presented as means (SDs) unless otherwise indicated. Fatigue was assessed on a 6pt NRS. Anxiety was assessed with the A-PROMIS (scores range from from 4–20, with higher scores representing greater anxiety). Depression was assessed with the D-PROMIS (scores range from 4–20, with higher scores representing greater depression). Perceived stress was assessed with the PSS (range of scores from 0 [no perceived stress] to 16 [maximal perceived stress]). State fear was assessed using the SUDS (100pt scale, higher number indicates greater fear). VR realism, presence, and involvement are from the iGroup Presence Questionnaire, for each domain represents the average score on a 0–6 Likert scale, where a higher number is increasing presence, involvement, or realism

^a^ Data were not normally distributed, Man-Whitney U tests were performed to assess between session differences

*p < 0.05

### Uphill steepness estimation

**Fig. 2:**
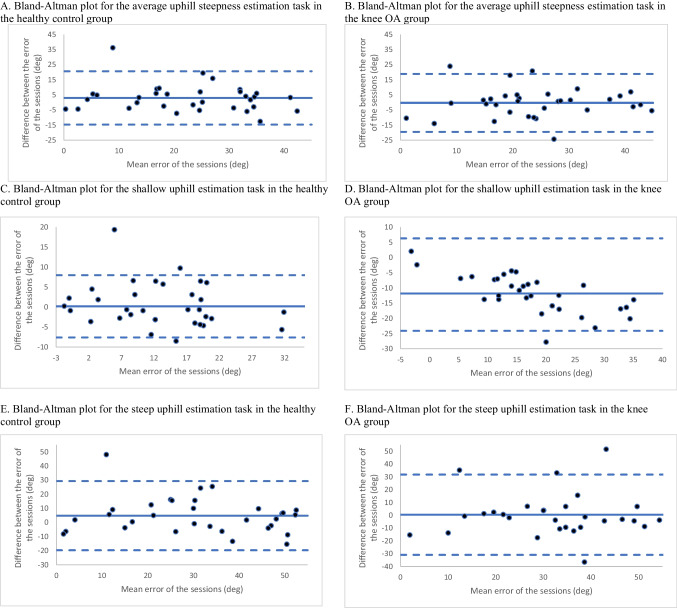
Bland–Altman plots for uphill steepness estimation tasks. For each plot, the solid line represents the mean of the difference between session one and two. The dotted lines are the upper and lower limits of agreement (± 2SD) of the differennce between sessions

Participants in both groups overestimated uphill steepness in both sessions (Table 2). A RM ANOVA (2 [group] × 2 [session] × 5[hill steepness]), found only a significant effect of steepness (F_4, 640_ = 77.118, *p* < 0.001, partial η^2^ = 0.33), where the amount of overestimation increased as actual hill steepness increased. The average uphill steepness task demonstrated good test–retest reliability in both groups, with ICC = 0.85 (95% CI 0.70 to 0.93) and ICC = 0.80 (95% CI 0.59 to 0.90) for the healthy control and knee OA groups, respectively. These findings were unchanged when considering shallow and steep hills in the healthy control group with ICC values of 0.89 (95% CI 0.78 to 0.95) and 0.85 (95% CI 0.70 to 0.93), respectively. In the knee OA group, the ICC values for shallow (ICC = 0.75, 95% CI 0.49 to 0.88) and steep hills (ICC = 0.72, 95% CI 0.43 to 0.86) also indicated good reliability, although the values were slightly lower than the healthy control group. Similarly, the Bland–Altman plot (Fig. 2A and B) supports adequate test re-test reliability for average hill steepness, as most points fall within 2 SD from the mean between-session difference. Similar results were found when considering shallow and steep hills in both groups (Fig. 2C-F).

**Table 2:** Error in estimations for perceptual measures in sessions 1 and 2

Perceptual measure	Session 1 Mean (SD)	Session 2 Mean (SD)
Healthy control group		
Average uphill steepness error (degrees)	24.0 (11.9)	21.0 (13.2)
Shallow uphill steepness error (degrees)	13.2 (8.26)	12.4 (9.45)
Steep uphill steepness error (degrees)	34.0 (16.5)	29.3 (18.3)
Average downhill steepness error (degrees)	10.9 (13.4)	8.24 (11.0)
Shallow downhill steepness error (degrees)	4.22 (12.3)	1.59 (11.9)
Steep downhill steepness error (degrees)	17.8 (15.2)	15.4 (11.9)
Average distance-on-hill (meters)	1.14 (2.60)	1.34 (3.99)
Knee OA group		
Average uphill steepness error (degrees)	24.0 (12.0)	24.3 (11.8)
Shallow uphill steepness error (degrees)	12.0 (7.77)	12.9 (7.46)
Steep uphill steepness error (degrees)	35.4 (17.1)	34.9 (16.9)
Average downhill steepness error (degrees)	17.1 (12.2)	15.5 (11.7)
Shallow downhill steepness error (degrees)	7.18 (10.5)	3.96 (7.21)
Steep downhill steepness error (degrees)	27.1 (16.4)	26.7 (17.0)
Average distance-on-hill (meters)	−0.08 (2.65)	0.30 (2.48)

The SRD for the average uphill steepness task was 17.7 degrees for the healthy control group and 18.9 degrees for the knee OA group. This represents from 21 to 27% of the total range of possible scores when considering overestimation alone (i.e., a maximum of 65 degrees of overestimation when considering a 25 degree hill). SRD scores were lower for both groups when considering only shallow hills (healthy control group = 7.99 degrees and knee OA group = 10 degrees), and larger when considering steep hills (healthy control group = 18.6 degrees, knee OA group = 24.9 degrees). Secondary analyses in the full knee OA sample (including those with unstable knee pain) had similar test–retest reliability and SRD results (Supplementary 2).

### Downhill steepness estimation

**Fig. 3:**
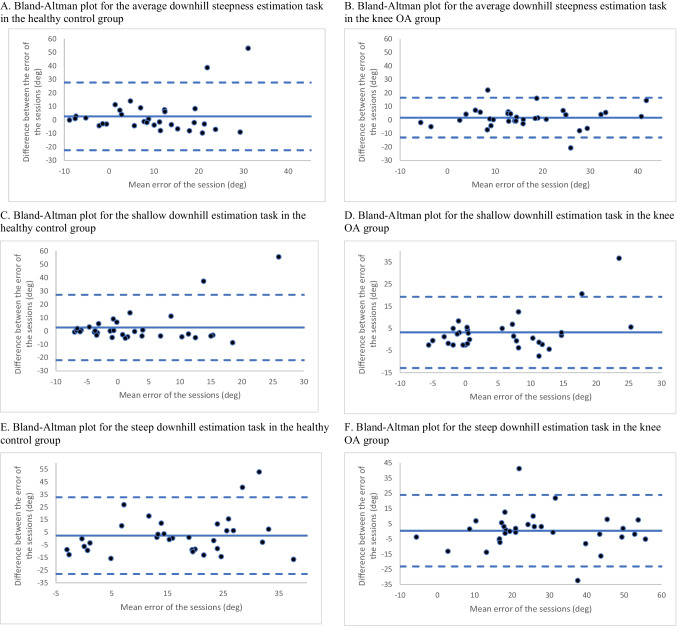
Bland–Altman plots for downhill steepness estimation tasks. For each plot, the solid line represents the mean of the difference between session one and two. The dotted lines are the upper and lower limits of agreement (± 2SD) of the differennce between sessions

Participants in both groups also overestimated downhill steepness, although their estimations were more accurate (less error) than for the uphill steepness task. A RM ANOVA found significant main effects of session (F_1, 640_ = 4.455, *p* = 0.035, partial η^2^ = 0.007, group (F_1, 640_ 0 = 31.543, *p* < 0.001, partial η^2^ = 0.05), and steepness (F_4, 640_ = 72.025, *p* < 0.001, partial η^2^ = 0.31). That is, as with uphill steepness, the amount of overestimation increased as actual hill steepness increased. Additionally, we found that the knee OA group overestimated downhill steepness compared to the healthy control group, and both groups had more accurate visuospatial perception (i.e., less overestimation) in session 2 relative to session 1. The ICC value for the downhill steepness task in the healthy control group was 0.37 (95% CI −0.28 to 0.69), which indicates poor test–retest reliability. Both the shallow (ICC = 0.45, 95% CI −0.11 to 0.73) and the steep (ICC = 0.32, 95% CI −0.39 to 0.66) hills had similarly poor reliability. The Bland–Altman plot for the healthy control group contained two outliers (points > 2SD from the mean between session differences) (Fig. 3A, C, E). In both cases, participants greatly overestimated hill steepness in the first session (mean error 58.0 degrees and 41.90 degrees) but had more accurate estimations in the second session (mean error 4.5 degrees and 2.2 degrees). A secondary analysis, where these two outliers were excluded, resulted in excellent reliability in average hill steepness estimation (ICC = 0.90, 95% CI 0.79 to 0.95) and good reliability for the shallow (ICC = 0.88, 95% CI 0.75 to 0.94) and steep (ICC = 0.77, 95% CI 0.52 to 0.89) hills. The RM ANOVA was also repeated with these outliers removed and results were consistent with the full sample (see Supplementary 2).

The ICC value for the knee OA group was 0.89 (95% CI 0.78 to 0.96), indicating good test–retest reliability. Similarly, both shallow and steep hills demonstrated good reliability in the knee OA group (shallow ICC = 0.74, 95% CI 0.47 to 0.87; steep ICC = 0.85, 95% CI 0.70 to 0.93). The Bland–Altman plots for the knee OA group demonstrates further evidence of good reliability, with good distribution of scores, and only two outliers despite narrow limits of agreement (Fig. 3B, D, F).

The SRD values for the knee OA group in the downhill steepness task was 14.7 degrees when considering average steepness, 12.9 degrees for shallow hills, and 17.7 degrees for steep hills. The SRDs in the healthy control group was comparatively large for all three hill types (average = 25.0 degrees, shallow = 21.2 degrees, steep = 41.6 degrees), although when the two outliers were removed, values were similar to the knee OA group (average = 12.1 degrees, shallow = 9.78 degrees, steep = 20.9 degrees). Secondary analyses in the full knee OA sample (including those with unstable knee pain) had similar test–retest and SRD results (Supplementary 2).

### Distance-on-hill

The distance-on-hill effect was tested by conducting a linear regression with slope (hill vs. flat) as a dummy coded dichotomous predictor under both a frequentist and Bayesian statistical framework. Neither the knee OA (Session 1: *B* = −0.09, *SE* = 1.23, 95% CI [−2.55, 2.38]*, p* = 0.95; Session 2: *B* = 0.30, *SE* = 1.49, 95% CI [−2.67, 3.28], *p* = 0.84) or healthy control (Session 1: *B* = 1.14, *SE* = 1.36, 95% CI [−1.56, 3.85]*, p* = 0.40; Session 2: *B* = 1.34, *SE* = 1.32, 95% CI [−1.30, 3.98]*, p* = 0.31) group showed a significant distance on hill effect in either session (via the frequentist analysis). The Bayesian analysis showed similar results for the knee OA (Session 1: *B* = −0.08, *EE* = 1.25, 95% CrI [−2.54, 2.36], *BF* = 3.12; Session 2: *B* = 0.31, *EE* = 1.51, 95% CrI [−2.65, 3.27], *BF* = 3.82) and healthy control (Session 1: *B* = 1.15, *EE* = 1.35, 95% CrI [−1.50, 3.81], *BF* = 4.88; Session 2: *B* = 1.34, *EE* = 1.34, 95% CrI [−1.35, 3.94], *BF* = 4.88) groups across sessions. Across both groups and sessions, the Bayes factors suggest that the data is ~ 3–5 times more likely under the model with slope (hill vs. flat) included. This favors there being a slight distance-on-hill effect, though this evidence is not especially strong as the Bayes factor evidence suggests this being only slightly (3–5 times) more likely than no effect. While credible intervals are not designed for hypothesis testing (Berger, 2006), they do provide interpretable estimates of uncertainty and can be viewed as a range of plausible effect sizes. For the knee OA group, the credible intervals ranged from −2.54 to 2.36 in session 1 and −2.65 to 3.27 in session 2, which suggests that the range of plausible values for the effect of slope (hill vs. flat) is roughly centered around zero with effect sizes of about 2–3 meters of error in either direction (under/over-estimation respectively) being plausible. For the healthy control group, the credible intervals ranged from −1.50 to 3.81 in session 1 and −1.35 to 3.94 in session 2, which suggests that the range of plausible values for the effect of slope (hill vs. flat) is roughly centered around 1 degree of (overestimation of hill compared to flat) error with effect sizes of about 1–2 meters of (underestimation of hill compared to flat respectively) error and 3–4 meters of (overestimation of hill compared to flat respectively) error being plausible.

Consistent with our *a priori* protocol, we averaged the distance-on-hill responses for all reliability analyses. The distance-on-hill task demonstrated poor reliability in both groups (healthy control: ICC = 0.29, 95% CI −0.44 to 0.65; knee OA: ICC = 0.38, 95% CI −0.22 to 0.68). Given that the distance-on-hill task score is a difference measure involving two separate distance estimations (and thus may be susceptible to the reliability paradox), we also conducted an off-protocol analysis to evaluate the reliability of the flat and the hill distance estimations separately. The ICC values for the flat estimation were excellent in both groups (healthy control ICC = 0.81, 95% CI 0.61 to 0.90: knee OA ICC = 0.82, 95% CI 0.64 to 0.90). There were very strong, positive associations between the two components of the distance-on-hill task (i.e., flat and hill distance estimations) in both groups (healthy control rho = 0.91, *p* < 0.001; knee OA rho = 0.87, *p* < 0.001).

**Fig. 4:**
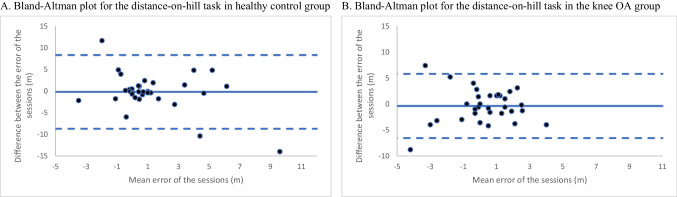
Bland–Altman plots for the distance-on-hill task. For each plot, the solid line represents the mean of the difference between session one and two. The dotted lines are the upper and lower limits of agreement (± 2SD) of the differennce between sessions

The Bland–Altman plot indicates that there may be a bias in distance-on-hill task responses in the healthy control group (Fig. 4A). Participants who showed a positive distance-on-hill effect (i.e., perceived distances as farther on hills than when that same distance was presented on the flat), also had large between-session error. Participants who showed minimal distance-on-hill effect (similar distance estimations regardless of environment, i.e., their mean error is close to zero), had little between-session error. The knee OA group did not demonstrate this bias, with no trends detected in visual analysis of the Bland–Altman plot (Fig. 4B). The SRD for the distance-on-hill task was 8.50 meters for the healthy control group and 6.20 meters for the knee OA group. These values are large when considering the small average distance-on-hill effect observed in both groups (healthy control session 1 = 1.14 meters, session 2 = 1.34 meters, knee OA session 1 = −0.08 meters, session 2 = 0.30 meters).

In terms of the components of the distance-on-hill task (i.e., the flat distance estimation and the hill distance estimation), the Bland–Altman plot of the flat distance estimation in the healthy control group (Supplementary 3) showed a similar bias as the distance-on-hill task, with participants who *underestimated* distance on a flat surface tending to have less between-session error than those who were more accurate. Again, this was bias not demonstrated in the knee OA group. The SRDs for flat distance estimation in the healthy control and knee OA groups were 7.78 meters and 9.02 meters, respectively. Similarly, the hill distance estimation had good–excellent test re-test reliability in both groups (healthy control: ICC = 0.75, 95% CI 0.49 to 0.88; knee OA: ICC = 0.84 95% CI 0.69 to 0.92). Visual analysis of Bland–Altman plots was consistent with this excellent reliability. The SRDs were 10.4 meters and 8.16 meters for the healthy control and knee OA groups, respectively. Secondary analyses in the full knee OA sample (i.e., also including those with unstable pain) had similar results.

## Discussion

We have undertaken the first comprehensive evaluation of test re-test reliability and smallest real difference (SRD) for three common measures of visuospatial perception in both a healthy cohort and a clinical pain (knee OA) cohort. Our results support the use of uphill and downhill steepness estimation tasks in research and clinical settings, showing good to excellent test re-test reliability in both groups, with high sensitivity to change, particularly in those with knee OA. However, the utility of the distance-on-hill task is uncertain: while test re-test reliability was poor, and there was low sensitivity to change (large SRDs relative to observed distance-on-hill effects), the base estimations for the task (flat and hill distance estimation) demonstrated excellent reliability. Taken together, our findings hold important practical and theoretical implications for embodied perception research.

We found good to excellent reliability of uphill and downhill steepness estimation tasks. This result was unchanged when we took the mean of the hill stimuli slopes, as well as only looking at shallow and steep hills, indicating that all hill stimuli are reliable. That both groups had similar results indicates that these tasks may be appropriate for both clinical and non-clinical samples, although further work is required in more diverse samples (i.e., other clinical groups and younger populations). Our results indicate that there does not appear to be a learning effect present in either group for the uphill steepness estimation task and that there may be a small learning effect in the downhill steepness estimation task given significantly less overestimation for both groups in session 2 relative to session 1. However, the latter effect is small, particularly when considering the SRD of downhill steepness estimation task. Thus, the current results support the use of both hill steepness estimation tasks in repeated-measures study designs, or to assess changes over time. While between group differences in hill steepness overestimation only partially replicated past work (downhill steepness differed between groups, uphill steepness did not), this was not a primary aim of the present study and may reflect an underpowered comparison.

Poor reliability for the distance-on-hill task may be underpinned by the so-called ‘reliability paradox’ (Hedge et al., 2018). Common in psychological research, this paradox can occur when tests use difference scores which have components that are highly correlated. In these cases, calculating a difference score effectively reduces the between-subject variability while preserving the measurement error (Hedge et al., 2018). This results in low ICC values, as the ICC is a relative measure of between-subject variability and measurement error. Given our experimental controls to reduce between-session variability were successful (e.g., no significant differences in process outcomes), it is less likely that clinical instability influenced reliability outcomes. Further, the strong correlations between flat and hill distance estimates, and that both the components of the distance-on-hill task (i.e., flat and hill distance estimation) had excellent reliability when assessed separately, supports this idea.

However, it is also important to consider that we found little evidence for a distance-on-hill effect in either population. Indeed, contrary to theories of embodied and action-specific perception (Witt, 2020) and previous work in healthy and clinical populations (Laitin et al., 2019a, b; Tenhundfeld & Witt, 2015), we observed only a small and non-significant distance-on-hill effect in the healthy control group, and no/smaller and non-significant distance-on-hill effects in the clinical population (who may be hypothesised to demonstrate a larger effect than the healthy group, due to their reduced body capacity). Importantly, such distance-on-hill findings are consistent with our previous research using the same tasks, but in a different knee OA population (*n* = 84) (MacIntyre et al., 2024).

We based our distance-on-hill task (VR based, same hill angle, similar target distances) on previous research which successfully induced the distance-on-hill effect in virtual reality and found high internal consistency (Laitin et al., 2019a, b). However, Laitin and colleagues used a visual-matching paradigm, where participants were required to match a ‘comparison cone’ to a previously viewed ‘target cone’, using a handheld controller to move the comparison cone either closer or farther away, until it visually matched the distance to the target cone. Instead, our study used a verbal estimation paradigm, where participants stated how far away target distances appeared to them when presented on flat surfaces and hills; partly chosen due to difficulties reported by older participants using the VR handheld controllers. Despite evidence of convergent validity for verbal and visual-matching versions of the distance-on-hill paradigm in real-life environments (Tenhundfeld & Witt, 2017), some researchers have theorised that verbal reports may introduce post-perceptual processes (e.g., response bias) to responses. While use of verbal reports may contribute to poor reliability of our distance-on-hill task by introducing additional measurement error (i.e., adding additional variability to responses), it seems unlikely that they fully explain poor reliability given that hill steepness estimation tasks also used verbal responses. Although, it is important to consider that hill steepness estimation anchors constrained the number of potential responses more so than the distance estimations (i.e., the latter task had a greater number of potential response options due to lack of anchors). Future work could address this issue by evaluating the test re-test reliability of a visual-matching distance-on-hill paradigm.

There was a range of SRD values, depending on hill slope and viewing angle (uphill vs. downhill). It is tempting to conclude that the lower SRD values of the shallow hills relative to steep hills mean that shallow hills may be more sensitive to change, and therefore the preferred task. However, it is important to consider that people were more accurate in their estimation of hill steepness of shallow hills compared to steep hills. Indeed, the average error for shallow hills ranged from 12.0 degrees to 13.2 degrees for uphill slopes, and 1.59 degrees to 7.18 degrees for downhill slopes. Thus, the shallow hill SRD values at times exceed the average error for shallow hills, indicating that a reduction in overestimation is unlikely to be detectable with the current tasks. Instead, steeper hills may be more relevant as the SRD values, while greater than shallow hills, represent a smaller proportion of the average error. Therefore, steeper hills may be more relevant to detect a change in hill steepness perception over time. Additionally, while the SRD represents the smallest detectable change given a task’s measurement properties, future work is required to determine the minimal clinical important difference of these tasks. The minimal clinical important difference of a task, not only encompasses change that is greater than measurement error, but also considers whether a change is clinically or behaviourally relevant (Anvari & Lakens, 2021). For example, given the theoretical links between visuospatial perception and behavioural outcomes (e.g., overestimation of hill steepness associated with behavioural avoidance (Proffitt, 2009)), understanding the change in hill steepness estimation that reflects a behavioural change (e.g., whether someone chooses to walk up the same hill) is an important next step.

Our use of a VR program to present spatial stimuli is consistent with contemporary research in the field (Creem-Regehr et al., 2019; Laitin et al., 2019a, b). VR is a powerful medium in perceptual research as it allows for systematic manipulation of various environmental characteristics (Tarr & Warren, 2002), with low participant and researcher burden. For example, hill attributes (steepness and distance), can be manipulated to explore their influence on perception, in a way that is not feasible in real world environments. Other potential confounders that are likely to influence spatial perception, such as ambient room temperature (Ekawati et al., 2023), can be controlled in virtual environments, thus reducing their influence on observed outcomes. One can also remove visual features that may influence estimation of steepness or distance, such as floor tiles and doorways. Despite these advantages, conducting visuospatial perception research in VR has limitations. Previous work has found biases in perceptual estimates in VR environments, specifically distances have been found to be systematically *underestimated* in VR environments (Creem-Regehr et al., 2015; Renner et al., 2013). This is consistent with our work, where both flat and hill distances were underestimated. The current work primarily evaluated the distance-on-hill task, which is a relative measure of distance error (both flat and hill distances were measured in VR), and therefore should have not been affected by systematic underestimation. However, underestimation of distances may have influenced results if there was differential scaling of flat and hill distance perception (e.g., distances on hills had less systematic underestimation than distances on the flat).

While the cause of VR distance underestimation is still debated, technological (e.g., field of view, head mounted display weight) (Buck et al., 2018; Kelly, 2022), environmental, and experiential (e.g., movement or body-based) (Creem-Regehr et al., 2023) factors are implicated. Embodiment may also play a role in the underestimation observed in virtual environments. When people are in VR, there are usually no visible virtual body parts unless a full-body avatar is rendered. Thus, they may be less influenced by energetic demands because of this disembodiment (Mohler et al., 2010) and they may underestimate because they do not have a body with which to ‘scale’ their judgments as they do in the real world (Proffitt et al., 2022). Indeed, people have more accurate VR-based distance estimation (i.e., less underestimation) when they embody an avatar than when they do not (Mohler et al., 2010; Phillips et al., 2010). Further, enhancing the level of embodiment by having the avatar track the participant’s movements resulted in greater accuracy when compared to a static avatar (Ries et al., 2008). The present study did not use an avatar, although we did employ head-tracking (e.g., visual field moved in time to head movements). Lack of embodiment and presence within VR may have influenced our results, therefore, future work should aim to improve the sense of presence and/or embodiment in the virtual world. Embodiment could be induced through use of an avatar, while presence could be improved from multisensory input (e.g., sound), or a longer familiarisation protocol where the participant can move freely within an interactive virtual world (Kilteni et al., 2012).

This study had several methodological strengths and limitations. Aside from age and gender, we did not collect data on sample demographics (e.g., education levels, ethnicity, race), therefore it is unclear whether our sample was representative. Additionally, we only evaluated VR presence (i.e., the sense of being embedded in the virtual world) in the healthy control group. It is possible that the two groups different in levels of VR presence, which may have influenced task reliability. After participant exclusions (e.g., for unstable pain), we did not achieve our *a priori* sample size. However, given that our sample size calculations were conservative (ICC = 0.4), we were likely still powered to detect poor task reliability (ICC ≤ 0.5). While we pre-registered testing of reliability in healthy volunteers, we did not pre-register testing in the knee OA group. However, that we largely replicated a pre-specified protocol, and clearly documented all deviations, allays serious concerns. Our knee OA sample was recruited via other experimental studies, and thus their participation in other research activities (e.g., completing an exercise task), may have influenced their responses in the follow-up session. However, the impact is likely minimal given that study activities were brief (30–45 min) and occurred a week prior to follow-up testing. We also measured several potential confounders that have previously been hypothesised to influence measures of spatial perception; however, there may have been other factors that influenced our results that we did not evaluate (e.g., blood glucose levels). Finally, as with all perceptual studies, our results may have been influenced by a response bias, where participants biased their estimations to conform to the research aims (Durgin et al., 2009). However, given the number of estimations (5–20 responses per task), and the time between sessions (~ 1 week), it is unlikely that participants were able to bias their responses in a way that was consistent with improving their reliability at the tasks.

## Conclusion

Virtual reality hill steepness estimation tasks are a promising measure of visuospatial perception – with excellent test re-test reliability and good sensitivity to change in clinical and healthy populations. However, the distance-on-hill task in the current study demonstrated poor test re-test reliability and low sensitivity to change, and therefore has limited experimental and clinical utility.

## Supplementary Information

Below is the link to the electronic supplementary material.

Supplementary file1 (DOCX 15 KB)

Supplementary file2 (DOCX 61 KB)

Supplementary file3 (DOCX 33 KB)

## Data Availability

De-identified data and an R Markdown PDF file for all analyses conducted in R are available on OSF (link: https://osf.io/btfsh/).

## Footnotes

1 Bayes factors were computed using bridge sampling and thus the reported values are approximations rather than direct calculations.
