# Supplementary material for: Assessing visuospatial perception in clinical and healthy populations: Test–retest reliability and smallest real difference of hill steepness estimation and the distance-on-hill task in virtual reality
Source: Psychol Res. 2025 May 20;89(3):101. doi: 10.1007/s00426-025-02125-0 (PMC12092535; doi:10.1007/s00426-025-02125-0)
Supplement: Supplementary file 1 — Supplementary file1 (DOCX 15 KB) [file 426_2025_2125_MOESM1_ESM.docx]

Guidelines for Reporting Reliability and Agreement Studies (GRRAS)

|  | Checklist item | Information reported (Yes/No) | Page # |
| --- | --- | --- | --- |
| TITLE AND ABSTRACT | Identify in the title of abstract that interrater/interrater reliability of agreement was investigated | Yes | 1 |
| INTRODUCTION | Name and describe the diagnosis of measurement device of interest explicitly | Yes | 4 |
|  | Specify the subject population of interest | Yes | 6 |
|  | Specify the rater population of interest (if applicable) | Yes (in methods) | 8 |
|  | Describe what is already knows about reliability and agreement and provide a rationale for the study (if applicable) | Yes | 4-5 |
| METHODS | Explain how the sample size was chosen. State the determined number of raters, subjects/objects, and replicate observations | Yes | 7 |
|  | Describe the sampling method | Yes | 7-8 |
|  | Describe the measurement/rating process (e.g., time interval between repeated measurements, availability of clinical information, blinding) | Yes | 8-9 |
|  | State whether measurements/ratings were conducted independently | Not applicable (single rater) |  |
|  | Describe the statistical analysis | Yes | 11-12 |
| RESULTS | State the actual number of raters and subjects/objects which were included and the number of replicate observations which were conducted | Yes | 8,12 |
|  | Describe the sample characteristics of raters and subjects (e.g., training experience) | Yes | 12, Table 1 |
|  | Report estimates of reliability and agreement including measures of statistical uncertainty | Yes | 13-15 |
| DISCUSSION | Discuss the practical relevance of results | Yes | 15 |
| AUXILIARY MATERIAL | Provide detailed results if possible (e.g., online) | Yes | Supp files 2 and 3 |
