# Supplementary material for: Assessing visuospatial perception in clinical and healthy populations: Test–retest reliability and smallest real difference of hill steepness estimation and the distance-on-hill task in virtual reality
Source: Psychol Res. 2025 May 20;89(3):101. doi: 10.1007/s00426-025-02125-0 (PMC12092535; doi:10.1007/s00426-025-02125-0)
Supplement: Supplementary file 2 — Supplementary file2 (DOCX 61 KB) [file 426_2025_2125_MOESM2_ESM.docx]

|  |  |  | Between session differences | | |
| --- | --- | --- | --- | --- | --- |
|  | Session 1  Mean (SD) | Session 2  Mean (SD) | p value | Cohen’s d | mean difference  95% CI |
| Age (years) | 66.8 (8.48) | |  |  |  |
| Gender | 21 females, 15 males | |  |  |  |
| Time of session | 10:42AM (1.59 hours) | 10:11AM (1.64 hours) | 0.01 | 0.44 | -0.51,  -0.92 to 0.11 |
| Fatigue (6pt NRS) | 1.97 (1.21) | 1.70 (1.31) | 0.13 | 0.21 | -0.27  -0.62 to 0.09 |
| Anxiety ^a^ | 5.84 (2.36) | 5.57 (2.27) | 0.21 | - | -0.27 |
| Depression ^a^ | 5.92 (3.12) | 5.65 (2.76) | 0.41 | - | -0.27 |
| Mood | 8.00 (1.65) | 7.89 (1.78) | 0.60 | 0.06 | -0.11  -0.52 to 0.31 |
| Average state fear | 24.5 (19.8) | 22.9 (15.7) | 0.40 | 0.09 | -1.6  -5.27 to 2.16 |
| 25deg hills state fear | 50.1 (34.4) | 52.4 (31.3) | 0.47 | 0.07 | 2.3  -4.07 to 8.68 |
| Pain intensity | 5.08 (1.90) | 4.68 (1.86) | 0.10 | 0.22 | -0.40  -0.89 to 0.08 |

Supplementary File 2 – knee OA group full sample analysis

Table 1: full sample knee OA group (n=36) demographics

Results are presented as means (SDs) unless otherwise indicated. Fatigue was assessed on a 6pt NRS. Anxiety was assessed with the A-PROMIS (scores range from from 4-20, with higher scores representing greater anxiety). Depression was assessed with the D-PROMIS (scores range from 4-20, with higher scores representing greater depression). Perceived stress was assessed with the PSS (range of scores from 0 [no perceived stress] to 16 [maximal perceived stress]). State fear was assessed using the SUDS (100pt scale, higher number indicates greater fear).

^a^ Data were not normally distributed, Man-Whitney U tests were performed to assess between session differences.

*p<0.05

Table 2: full sample knee OA group (n=36) ICC and SRD for each task

| **Task** | **ICC value (95% CI)** | **SRD value** |
| --- | --- | --- |
| Average uphill steepness | 0.82 (0.65 – 0.91) | 19.0 degrees |
| Shallow uphill steepness | 0.74 (0.49 – 0.87) | 13.4 degrees |
| Steep uphill steepness | 0.75 (0.51 – 0.87) | 31.3 degrees |
| Average downhill steepness | 0.90 (0.81 – 0.95) | 14.7 degrees |
| Shallow downhill steepness | 0.83 (0.66 – 0.91) | 16.1 degrees |
| Steep downhill steepness | 0.86 (0.0.72- 0.93) | 23.5 degrees |
| Distance-on-hill | 0.38 (-0.22 – 0.68) | 6.20 meters |
| Flat distance | 0.84 (0.69 – 0.92) | 9.02 meters |
| Hill distance | 0.82 (0.64 – 0.91) | 8.16 meters |

Figure 1: BA plots for each task

| A. Average uphill steepness | B. Shallow uphill steepness |
| --- | --- |
|  |  |
| C. Steep uphill steepness | D. Average downhill steepness |
|  |  |
| E. Shallow downhill steepness | F. Steep downhill steepness |
|  |  |
| G. Distance-on-hill | H. Flat distance |
|  |  |
| I. Hill distance |  |
|  |  |
