# Supplementary material for: Assessing visuospatial perception in clinical and healthy populations: Test–retest reliability and smallest real difference of hill steepness estimation and the distance-on-hill task in virtual reality
Source: Psychol Res. 2025 May 20;89(3):101. doi: 10.1007/s00426-025-02125-0 (PMC12092535; doi:10.1007/s00426-025-02125-0)
Supplement: Supplementary file 3 — Supplementary file3 (DOCX 33 KB) [file 426_2025_2125_MOESM3_ESM.docx]

Supplementary file 3: Bland-Altman plots for distance estimation on the flat and on the hill

Figure 1: Bland-Altman plots for the flat and hill components of the distance-on-hill task. For each plot, the solid line represents the mean of the difference between session one and two. The dotted lines are the upper and lower limits of agreement (+/- 2SD) of the differennce between sessions

| A. Bland-Altman plot for flat distance estimation in the healthy control group | B. Bland-Altman plot for flat distance estimation in the knee OA group |
| --- | --- |
| C. Bland-Altman plot for hill distance estimation in the healthy control group | D. Bland-Altman plot for hill distance estimation in the knee OA group |
